# The efficacy of semi-quantitative urine protein-to-creatinine (P/C) ratio for the detection of significant proteinuria in urine specimens in health screening settings

**DOI:** 10.1186/s40064-016-3389-5

**Published:** 2016-10-13

**Authors:** Chih-Chun Chang, Ming-Jang Su, Jung-Li Ho, Yu-Hui Tsai, Wei-Ting Tsai, Shu-Jene Lee, Tzung-Hai Yen, Fang-Yeh Chu

**Affiliations:** 1 Department of Clinical Pathology, Far Eastern Memorial Hospital, No. 21, Sec. 2, Nanya S. Road, Banqiao, New Taipei City, Taiwan, ROC; 2 Division of Nephrology and Clinical Toxicology, Chang Gung Memorial Hospital, Lin-Kou Medical Center, Taoyuan, Taiwan, ROC; 3 School of Medical Laboratory Science and Biotechnology, Taipei Medical University, Taipei, Taiwan, ROC; 4 Graduate School of Biotechnology and Bioengineering, Yuan Ze University, Taoyuan, Taiwan, ROC; 5 Department of Medical Laboratory Science and Biotechnology, Yuanpei University of Medical Technology, Hsinchu, Taiwan, ROC

**Keywords:** Chronic kidney disease, Health screening, Protein-to-creatinine ratio, Proteinuria

## Abstract

**Background:**

Urine protein detection could be underestimated using the conventional dipstick method because of variations in urine aliquots. This study aimed to assess the efficacy of the semi-quantitative urine protein-to-creatinine (P/C) ratio compared with other laboratory methods.

**Methods:**

Random urine samples were requested from patients undergoing chronic kidney disease screening. Significant proteinuria was determined by the quantitative P/C ratio of at least 150 mg protein/g creatinine. The semi-quantitative P/C ratio, dipstick protein and quantitative protein concentrations were compared and analyzed.

**Results:**

In the 2932 urine aliquots, 156 (5.3 %) urine samples were considered as diluted and 60 (39.2 %) were found as significant proteinuria. The semi-quantitative P/C ratio testing had the best sensitivity (70.0 %) and specificity (95.9 %) as well as the lowest underestimation rate (0.37 %) when compared to other laboratory methods in the study. In the semi-quantitative P/C ratio test, 19 (12.2 %) had positive, 52 (33.3 %) had diluted, and 85 (54.5 %) had negative results. Of those with positive results, 7 (36.8 %) were positive detected by traditional dipstick urine protein test, and 9 (47.4 %) were positive detected by quantitative urine protein test. Additionally, of those with diluted results, 25 (48.1 %) had significant proteinuria, and all were assigned as no significant proteinuria by both tests.

**Conclusions:**

The semi-quantitative urine P/C ratio is clinically applicable based on its better sensitivity and screening ability for significant proteinuria than other laboratory methods, particularly in diluted urine samples. To establish an effective strategy for CKD prevention, urine protein screening with semi-quantitative P/C ratio could be considered.

## Background

The prevalence of chronic kidney disease (CKD) has been increasing worldwide in recent decades. CKD in early stage is characterized by an increased glomerular filtration rate (GFR) with elevated urinary protein excretion. Therefore, protein detection in urine has been widely performed as part of the laboratory diagnosis in early CKD screening. After being diagnosed with early CKD, a patient could be evaluated and treated quickly and appropriately in accordance with current guidelines for CKD (Levey et al. 2007; Stevens and Levin 2013).

The gold standard for measuring urine protein has been 24-h urine protein excretion. Nevertheless, this method is notorious for its inconvenience and the inaccuracy involved in collecting the 24-h urine sample. The most common laboratory test for proteinuria detection is urine dipstick. However, the detection results of urine protein could be underestimated because of the variation in urine samples. The concentration, or dilution due to excessive liquid consumption like water ingestion before the test, is one of the main factors affecting the variation of urine samples. To solve this problem, the urinary creatinine level was measured, and the urine protein-to-creatinine (P/C) ratio was calculated to correct for the variation in urine samples (Pugia et al. 2001; Wallace et al. 2001). The P/C ratio was shown to have acceptable sensitivity and specificity compared with the standard 24-h urine protein testing. Recently, a novel semi-quantitative dipstick test for the urine P/C ratio has become available for efficiently detecting proteinuria (Watanabe et al. 2005; Guy et al. 2009). It was also demonstrated that the urine P/C ratio evaluated by the semi-quantitative method could be practicable and reliable in the health screening for CKD (Wang et al. 2009).

In Taiwan, the government has subsidized regular physical check-ups for people elder than 40 years old, and the exams are annual for individuals older than 65 years and triennial for those aged between 40 and 64 years old. Because of the high prevalence of CKD, urine protein detection was incorporated to identify patients at risk of early stage nephropathy in the physical check-up program. However, the program did not define the optimal laboratory method for detecting proteinuria. As the consequence, the detection rate of significant proteinuria might be different and incomparable between screening centers. Hence, the aim of this study is to evaluate and compare the efficacy of different methods for the detection of significant proteinuria, especially in diluted urine specimens.

## Methods

A total of 2932 subjects aged at least 40 years and participating in regular physical examinations, subsidized by the National Health Insurance Administration, Ministry of Health and Welfare, Taiwan, were enrolled after the written informed consent were obtained from the participants or one member of their family. Random mid-stream urine was collected for each subject in this study. Urine samples were then sent to Department of Clinical Pathology, Far Eastern Memorial Hospital, for further laboratory analysis. This investigation was approved by the research ethics review committee of Far Eastern Memorial Hospital and was supervised by the data safety monitoring board.

**Fig. 1:**
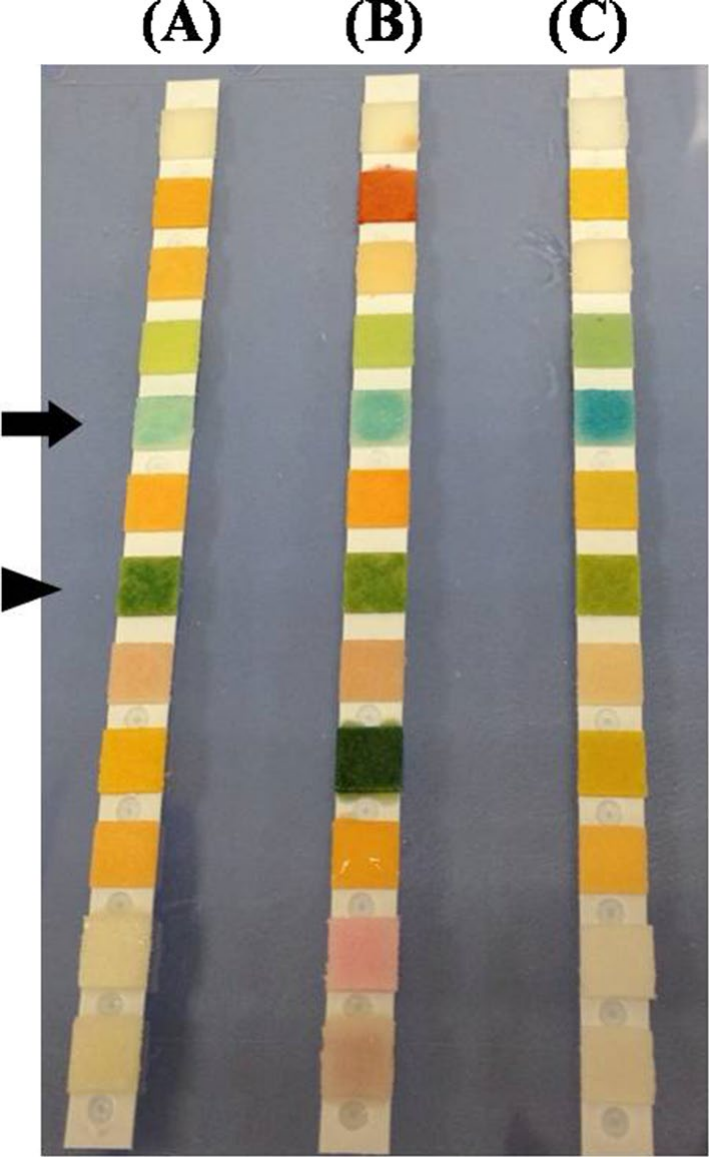
The commercially available dipstick test (Clinitek Atlas^®^PRO™12 Reagent Pak) for the semi-quantitative urine protein-to-creatinine (P/C) ratio. In addition to a traditional dipstick testing pad for color, pH, protein, glucose, ketone, bilirubin, urobilinogen, nitrite, blood and leukocyte esterase, the novel dipstick test incorporated a relatively low-protein pad (*arrow*) and creatinine pad (*arrowhead*) for the automated calculation of semi-quantitative urine P/C ratio. **a** A urine strip with normal P/C ratio (less than 150 mg protein/g creatinine), **b** a urine strip with abnormal P/C ratio (within the range of 150–299.9 mg protein/g creatinine) and **c** a urine strip with abnormal P/C ratio (within the range of 300–499.9 mg protein/g creatinine)

Each urine sample underwent a dipstick test using a commercially available dipstick test (Clinitek Atlas^®^PRO™12 Reagent Pak, Siemens, Indiana, USA), as shown in Fig. 1, in an automated urine chemistry analyzer (Clinitek Atlas, Siemens, Indiana, USA) routinely used in the local laboratory. The automated urine testing system included a traditional dipstick protein testing pad and a relatively new low-protein pad incorporating semi-quantitative creatinine pad to correct for the possible effect of dilution and concentration of the urine sample upon urine protein detection. The reports of the semi-quantitative urine P/C ratio were presented as normal, positive at increasing ordinal levels of 150, 300, and >500 mg of protein per gram of creatinine (mg protein/g creatinine), as well as diluted (which required re-collection and re-test). The result of the traditional protein pad was recorded simultaneously. The quantitative urine protein and creatinine concentrations were determined by an automated chemistry analyzer (Hitachi 911, Roche, Minnesota, USA), in which the former was measured by a turbidimetric method (U/CSF Protein, Roche, Minnesota, USA) and the latter was measure by Jaffe’s method (Crea, Roche, Minnesota, USA). The quantitative urine P/C ratio was then calculated. All laboratory methods were performed according to standard operating procedures. Significant proteinuria was considered as the quantitative urine protein level of at least 30 mg/dL or the quantitative P/C ratio of at least 150 mg protein/g creatinine (Stevens and Levin 2013).

Using the quantitative P/C ratio as the gold standard, the performance of different methods for detecting the existence of significant proteinuria, including traditional semi-quantitative dipstick protein, the new semi-quantitative dipstick P/C ratio, and quantitative urine protein level, was evaluated. Underestimation was defined as the failure of a test to identify significant proteinuria of at least 150 mg protein/g creatinine detected by the standard method of the quantitative P/C ratio. Additionally, urine aliquots with a creatinine concentration of less than 50 mg/dL were regarded as diluted.

Statistical analyses were performed by computing the sensitivity, specificity, positive predictive value (PPV), negative predictive value (NPV) and Kappa coefficient of agreement for each laboratory method. The statistical software, SPSS (version 19.0; SPSS Inc., Chicago, USA), was used for all analyses.

## Results

Table 1 shows the correlation between the semi-quantitative and quantitative urine P/C ratios. Excluding the urine samples with diluted results by the semi-quantitative P/C ratio, the sensitivity, specificity, PPV, NPV and Kappa coefficient for the semi-quantitative P/C ratio to detect significant proteinuria of at least 150 mg protein/g creatinine were 75.6 and 95.9 %, 0.72, 0.96 and 0.52, respectively (Table 4). Furthermore, the sensitivity, specificity, PPV, NPV and Kappa coefficients for the semi-quantitative P/C ratio including the urine samples with dilution results were 70.0, 95.9 %, 0.75, 0.95 and 0.56, respectively. The rate of underestimation was 0.37 % for the semi-quantitative P/C ratio to detect significant proteinuria.

**Table 1:** Correlation of the urine protein-to-creatinine (P/C) ratio results between the semi-quantitative and quantitative methods

	Quantitative P/C ratio (mg protein/g creatinine)			
	<150	150–299.9	300–499.9	>500
Semi-quantitative P/C ratio (mg protein/g creatinine)				
Normal	2356	103	5	1
150	88	125	19	5
300	13	33	31	25
>500	1	4	3	68
Diluted	27	18	6	1

The correlation between the traditional dipstick urine protein test and quantitative urine P/C ratio is shown in Table 2. The sensitivity, specificity, PPV, NPV and Kappa coefficient were calculated to be 45.0, 98.3 %, 0.83, 0.91 and 0.37, respectively (Table 4). Table 3 shows the correlation between the quantitative urine protein and P/C ratio. The sensitivity, specificity, PPV, NPV and Kappa coefficients for the quantitative urine protein were calculated to be 50.1, 98.2 % 0.84, 0.92 and 0.40, respectively (Table 4). Moreover, the underestimation rate for the dipstick and quantitative urine protein tests were 8.39 and 7.61 %, respectively.

**Table 2:** Correlation between the dipstick urine protein and quantitative urine protein-to-creatinine (P/C) ratio results

	Quantitative P/C ratio (mg protein/g creatinine)			
	<150	150–299.9	300–499.9	>500
Dipstick protein				
Negative	2443	209	28	9
1+	39	62	25	16
2+	3	12	11	50
3+	0	0	0	25

**Table 3:** Correlation between the quantitative urine protein and quantitative urine protein-to-creatinine (P/C) ratio results

	Quantitative P/C ratio (mg protein/g creatinine)			
	<150	150–299.9	300–499.9	>500
Quantitative protein level (mg/dL)				
< 30	2441	191	28	4
30–64.9	43	85	27	28
65–199.9	1	7	9	52
>200	0	0	0	16

**Table 4:** Comparison of the sensitivity, specificity, positive and negative predictive values, Kappa coefficient and rate of underestimation for each laboratory method with the quantitative urine protein-to-creatinine (P/C) ratio

Testing	Sen (%)	Spe (%)	PPV	NPV	Kappa	Und (%)
Semi-quantitative P/C ratio (excluding diluted samples)	75.6	95.9	0.72	0.96	0.52	0.37
Semi-quantitative P/C ratio (including diluted samples)	70.0	95.9	0.75	0.95	0.56	0.37
Dipstick protein	45.0	98.3	0.83	0.91	0.37	8.39
Quantitative protein	50.1	98.2	0.84	0.92	0.40	7.61

*Sen* sensitivity, *Spe* specificity, *PPV* positive predictive value, *NPV* negative predictive value, *Kappa* Kappa coefficient, *Und* rate of underestimation

Importantly, we further found that a total of 156 urine samples were considered as diluted, accounting for 5.3 % of the 2932 urine aliquots. Significant proteinuria was found in 60 (39.2 %) of these diluted samples. In the semi-quantitative P/C ratio test, 19 (12.2 %) had positive, 52 (33.3 %) had diluted, and 85 (54.5 %) had negative results. The screening rate of the semi-quantitative P/C ratio test was 45.5 %, including the positive and diluted results. For the 19 subjects with positive results of the semi-quantitative P/C ratio test, 7 (36.8 %) were positive for the traditional dipstick urine protein test, and 9 (47.4 %) were positive for the quantitative urine protein test. Additionally, 52 had diluted results of the semi-quantitative P/C ratio test and 25 (48.1 %) of these subjects had significant proteinuria of at least 150 mg protein/g creatinine. Of these, all were assigned as no significant proteinuria by both traditional dipstick test and quantitative urine protein test.

## Discussions

The prevalence of CKD is increasing, and the condition is becoming a global public health problem. Taiwan is one of countries with high prevalence of CKD. The national prevalence of CKD was estimated to be 11.93 %, and only 3.54 % of patients in Taiwan with CKD were aware of their disorder (Wen et al. 2008). If not diagnosed early and well controlled, CKD might progress into end-stage renal disease (ESRD). Patients with ESRD usually received kidney transplantation or hemodialysis therapy, both of which cost disproportionate amounts in healthcare resources and impaired the quality of life of patients. According to the report of the National Health Insurance Administration, Ministry of Health and Welfare, Taiwan, in 2008, approximately 56,000 people (0.24 % of all people covered by the National Health Insurance) underwent regular dialysis treatment and expended 11 % of the annual healthcare reimbursement. Therefore, it is important for healthy individuals to receive periodic urine screening to prevent the development of incident CKD.

The method for screening for significant proteinuria has not been clearly defined and regulated in Taiwan. The relatively new method of protein detection on a urine dipstick incorporating urine creatinine estimation has been demonstrated to be a more sensitive indicator for detecting significant proteinuria (Xin et al. 2004; Price et al. 2005; Wang et al. 2009; Methven et al. 2010). Distinct from the conventional dipstick test, the semi-quantitative urine P/C ratio possessed an additional advantage for the correction of urine concentration variation by detecting urine creatinine level and, therefore, increased the screening rate of diluted urine samples. In our institution, the abnormal or diluted results of semi-quantitative urine P/C ratio testing for urine samples sent to the laboratory were further interpreted and commented by the clinical pathologist, and were subsequently validated in real time in the outpatient clinic or in the ward. However, the traditional dipstick test remains the most common method used by clinical laboratories for detecting significant proteinuria. As a result, physicians should correlate the clinical manifestations of patients with the urinalysis report carefully because significant proteinuria might be missed because of diluted or underestimated urine samples in such situations. Therefore, it is necessary that the efficacy of each laboratory methods for detecting proteinuria should be compared and analyzed. To the best of our knowledge, our study is the first to describe the underestimated report of urine protein examination for the semi-quantitative P/C ratio, traditional dipstick and quantitative urine protein methods.

The benefits of routine screening for CKD remain controversial, in spite of the increasing prevalence of CKD. Previously, it was suggested that there was no cost-effectiveness of early detection of urine protein for retarding the progression and decreasing the mortality of CKD, unless the urine protein examination was selectively directed toward high-risk groups such as elderly and hypertensive subjects (Boulware et al. 2003). Otherwise, urine protein examinations were recommended to be conducted at an intermission of approximately 10 years in the healthy adult population (Boulware et al. 2003). However, a recent study revealed that universal screening for proteinuria appeared to be an effective strategy for reducing the CKD population because a high positive rate of proteinuria in the general population was shown in Asians, even in persons without hypertension or diabetes (Yamagata et al. 2008). In view of this fact, a more sensitive urine screening tool for proteinuria is necessary, especially for Asian subjects. Besides, the average costs of the quantitative and semi-quantitative urine P/C ratio for a single test in our institution were 2.58 and 2.42 US dollars, respectively. It seems that use of the semi-quantitative urine P/C ratio testing could lead to a minor decrease in cost compared to that of the gold standard testing. The semi-quantitative urine P/C ratio might be a good choice for early detection of proteinuria and appeared to be more effective than the traditional dipstick urine protein test, particularly in diluted urine aliquots.

It is important for physicians to screen for and diagnose CKD based on rapid and accurate urine examinations. Therefore, patients are required to be available for re-collection of urine aliquots for repeated urinalysis, consuming unnecessary laboratory and time costs for further testing confirmation. Considering these factors, patients should be well educated to not consume too much water before urine sample collection because normal or diluted results of the semi-quantitative urine P/C ratio would be present, and falsely negative results could be reported. If this factor is taken into consideration, unnecessary urinalysis retests could be avoided, and timely management could be initiated as needed.

There are some limitations in the present study. First, it was unknown whether subjects undergoing urine protein screening had any underlying diseases such as hypertension or diabetes. Moreover, information regarding racial differences, family history and personal medication use were not available. Additionally, the age of the subjects for proteinuria screening was not included in the exclusion criteria in our study, resulting in some extent of selection bias.

To summarize, the present study indicated that the urine P/C ratio evaluated by a semi-quantitative method is clinically applicable based on its better sensitivity and screening ability for significant proteinuria than other laboratory methods, particularly in diluted urine samples. To establish an effective strategy for CKD prevention in the Asian populations, urine protein screening with semi-quantitative P/C ratio could be considered.
